# Retrospective Analysis of Cushing's Disease with or without Hyperprolactinemia

**DOI:** 10.1155/2014/919704

**Published:** 2014-11-23

**Authors:** Cheng Huan, Chao Lu, Guang-ming Xu, Xin Qu, Yuan-ming Qu

**Affiliations:** ^1^Department of Neurosurgery, Shandong Provincial Hospital Affiliated to Shandong University, Jinan 250000, China; ^2^Department of Laboratory Medicine, Shandong Provincial Hospital Affiliated to Shandong University, Jinan 250000, China; ^3^Department of Intensive Care Unit, Shandong Provincial Hospital Affiliated to Shandong University, Jinan 250000, China

## Abstract

*Objective*. We compared the characteristics of patients with Cushing's disease alone with those of patients with Cushing's disease and hyperprolactinemia.* Methods*. Eighty-four patients were enrolled between 2002 and 2011, in a hospital in China. Clinical, endocrinological, and histopathological data, MRI scans, and surgical outcomes were reviewed throughout the follow-up period.* Results*. Patients with Cushing's disease and hyperprolactinemia had a younger age at diagnosis (30.28 ± 14.23 versus 36.08 ± 10.91 years; *P* = 0.037) and a larger adenoma maximal diameter (2.44 ± 1.32 versus 1.44 ± 1.05 cm; *P* < 0.001) than patients with Cushing's disease alone. Menstrual disorders (*P* = 0.027) and visual field defects (*P* = 0.021) were more common and progressive obesity (*P* = 0.009) and hypertension (*P* < 0.001) were less common in patients with Cushing's disease and hyperprolactinemia. The rate of normalization of hormonal levels was lower (41.7% versus 91.7%; *P* < 0.001) and the recurrence rate was higher (36.1% versus 8.3%; *P* < 0.001) in patients with Cushing's disease and hyperprolactinemia.* Conclusions*. Careful long-term follow-up is needed of patients with Cushing's disease and hyperprolactinemia.

## 1. Introduction

The most common etiology of spontaneous excess cortisol production is Cushing's disease due to hypersecretion of pituitary adrenocorticotropic hormone (ACTH) by a corticotroph adenoma [1]. Cushing's disease is a rare disorder, with an annual incidence of 1–3 per million and a female : male ratio of 3 : 1, that is responsible for significant morbidity and mortality resulting from cardiovascular complications, infections, and psychiatric disturbances [2]. It is now recognized that, in a small proportion of cases, Cushing's disease is accompanied by an elevated prolactin (PRL) level. For example, Lado-Abeal and colleagues identified a mildly elevated serum PRL in 20% of women with Cushing's disease and menstrual abnormalities [3], and both Mahler et al. [4] and Sherry et al. [5] have presented a patient with Cushing's disease and symptomatic hyperprolactinemia. Although Cushing's disease with hyperprolactinemia due to mixed ACTH- and PRL-secreting adenomas occurs rarely, elevated preoperative PRL levels in Cushing's disease are of diagnostic significance. Despite the abundance of published data concerning various aspects of the clinical course and treatment of Cushing's disease, very few investigations have focused on the characteristics of patients with Cushing's disease and hyperprolactinemia, and there is a paucity of studies comparing the clinical features of patients with Cushing's disease and hyperprolactinemia and those with Cushing's disease but no hyperprolactinemia. This is particularly the case for patients in China, about whom very little information is available concerning clinical outcomes following transsphenoidal surgery.

The aims of our investigation were to describe the prevalence and clinical features of Cushing's disease in patients treated with transsphenoidal surgery at our hospital in China and to evaluate the differences between patients with Cushing's disease and hyperprolactinemia and those with Cushing's disease alone. We therefore performed a retrospective analysis of patients with Cushing's disease, with or without concomitant hyperprolactinemia, to clarify the clinical features and confirm the efficacy of transsphenoidal surgery for treatment of the disease. This study provides novel insights that extend our current knowledge and will help to improve the diagnosis and treatment of patients with Cushing's disease and hyperprolactinemia.

## 2. Materials and Methods

### 2.1. Patients

This was a retrospective study of patients diagnosed with Cushing's disease, who had been treated by primary transsphenoidal surgery between January 2002 and June 2011, at the Department of Neurosurgery, Shandong Provincial Hospital Affiliated to Shandong University, Jinan, Shandong, China. The diagnosis of Cushing's disease was made on the basis of the medical history, symptoms, the levels of serum hormones, immunohistochemical staining, computer-aided tomographic (CT) imaging, and magnetic resonance imaging (MRI). Patients were divided into two groups, according to the preoperative hormonal levels and immunohistochemical results: a CD group (patients with elevated cortisol only) and a CD + PRL group (patients with both elevated cortisol and PRL levels). Due to the retrospective nature of this study, written informed consent was deemed not to be required. Oral informed consent was obtained from each participant, and the data were analyzed anonymously. The Ethics Committee of the Shandong Provincial Hospital Affiliated to Shandong University approved the research protocols.

Patients were included in the study if all the following criteria were satisfied: classical signs and symptoms of Cushing's disease; increased serum cortisol levels; loss of diurnal rhythm of serum cortisol; increased 24-hour urinary free cortisol levels; normal or slightly elevated plasma ACTH levels; lack of suppression of urinary free cortisol and serum cortisol following oral 2 mg dexamethasone loading; suppression of urinary free cortisol and serum cortisol after oral 16 mg dexamethasone (urinary free cortisol and/or serum cortisol suppressed by >50% of the baseline value following oral administration of 2 mg dexamethasone every 6 hours for 48 hours); an adenoma staining positively for ACTH; the presence of a pituitary mass on MRI; treatment by surgery alone; and availability of a complete set of medical records. An additional inclusion criterion required for the CD + PRL group was an adenoma staining positive for PRL.

The exclusion criteria for the CD group were patients with hypersecretion of two or more hormones; adenomas with positive immunohistochemical staining for two or more hormones; adrenal-derived Cushing's syndrome; ectopic Cushing's syndrome; and other potential causes of an elevated cortisol level. The exclusion criteria for the CD + PRL group were patients with hypersecretion of two or more hormones (except for cortisol and PRL); adenomas with positive immunohistochemical staining for two or more hormones (except for ACTH and PRL); and other causes of elevated cortisol and PRL levels.

Microscopically assisted transsphenoidal pituitary tumor resection surgery was performed by two experienced neurosurgeons (Yuan-ming Qu and Guang-ming Xu), and the resection was considered subtotal or complete in all enrolled patients. In order to accurately assess the effects of surgery, only patients treated by surgery alone were included in this study.

### 2.2. Data Extracted from the Medical Records

The data collected included the age and gender of the patient, past medical history, clinical presentation, preoperative neurological, endocrine, and ophthalmological status, and postoperative outcomes. The time from the onset of symptoms and signs to the date of diagnosis (time to diagnosis) was noted.

### 2.3. Investigations

**Figure 1:**
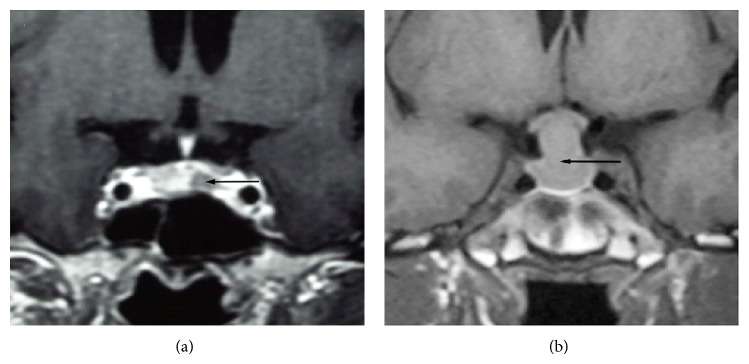
Representative T1-weighted coronal MRI scans. (a) Microadenoma (arrow). (b) Macroadenoma expanding into the suprasellar cistern (arrow).

The tumor size was measured using MRI. A microadenoma was defined as a tumor with a diameter of 10 mm or less, whereas a macroadenoma was defined as a tumor with a diameter of more than 10 mm [6]. Representative T1-weighted coronal MRI scans showing an example of a microadenoma and a macroadenoma are shown in Figure 1. Cavernous sinus invasion was suspected when adenoma tissue was located lateral to the cavernous portion of the internal carotid artery on MRI. Definite diagnosis could be made when the medial wall of the cavernous sinus was found to be destroyed during surgery by invasion [7]. The Knosp grade from the radiology findings was classified for degree of invasion. Pituitary imaging was also performed three months after surgery.

Endocrinological assessment was carried out in all patients, including measurements of the serum levels of cortisol, PRL, growth hormone (GH), thyroid stimulating hormone (TSH), luteinizing hormone (LH), follicle stimulating hormone (FSH), testosterone, estradiol, thyroxine, and progesterone. In this study, cortisol was measured using an electrochemiluminescent immunoassay kit (Roche Diagnostics GmbH, Mannheim, Germany) with a functional sensitivity of <8.5 nmol/L and intra-assay and interassay variation coefficients of <10% and <15%, respectively. The normal range for cortisol is 171–536 nmol/L. The serum level of PRL was measured using a commercially available chemiluminescence kit (Beckman Coulter, Inc., Brea, CA, USA). The normal range for PRL is 2.64–13.13 ng/mL in males, 3.34–26.72 ng/mL in premenopausal females, and 2.74–19.64 ng/mL in postmenopausal females. The analytical sensitivity for PRL was 0.25 ng/mL, and the intra-assay and interassay variation coefficients were <10% and <15%, respectively. Serum levels of cortisol and PRL were evaluated in all patients on three occasions: 3 days after surgery, approximately 3 months after surgery, and 12 months after surgery.

**Figure 2:**
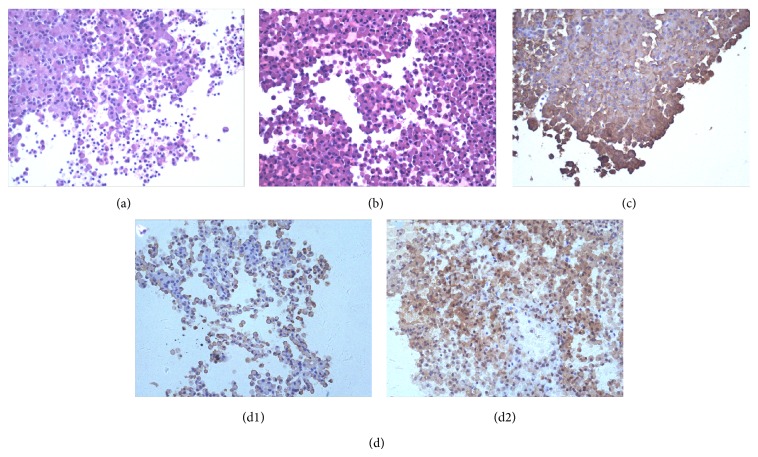
Surgical pathology analyses of the tumors. (a) H&E staining of tumor tissue from a patient in the CD group. (b) H&E staining of tumor tissue from a patient in the CD + PRL group. (c) Immunohistochemistry: cells of the adenoma from a patient in the CD group were immunoreactive to antibodies against ACTH. (d) Immunohistochemistry: cells of the adenoma from a patient in the CD + PRL group were immunoreactive to antibodies against ACTH (d1) and PRL (d2).

The resected tumor fragments were fixed in 10% buffered formalin, stained with hematoxylin and eosin (H&E) using routine histochemical methods, and embedded in paraffin blocks. Immunohistochemical analysis was also performed on specimens, using specific antibodies against ACTH (rabbit polyclonal antibody; Maxin Co. Ltd., Fujian, China), GH (rabbit polyclonal antibody; Maxin Co. Ltd., Fujian, China), PRL (rabbit polyclonal antibody; Maxin Co. Ltd., Fujian, China), FSH (mouse monoclonal antibody; Maxin Co. Ltd., Fujian, China), LH (mouse monoclonal antibody; Maxin Co. Ltd., Fujian, China), and TSH (mouse monoclonal antibody; Maxin Co. Ltd., Fujian, China). An Olympus BX53 upright fluorescence microscope (Olympus Co. Ltd., Shinjuku, Japan) was used to view and record the images. Examples of images obtained using these pathological techniques are shown in Figure 2 (original magnification ×400).

### 2.4. Postoperative Complications

Postoperative complications were divided into major and minor categories. A major complication was considered to be a disease or injury diagnosed after surgery that was potentially fatal and/or resulted in a permanent defect. Less serious adverse events, including diabetes insipidus (DI) and hyponatremia, were counted as minor complications. Urine volume measurements and serum electrolyte analyses were carried out for all patients. Serum levels of sodium were measured immediately before surgery and on days 1, 3, and 7 after surgery; hyponatremia was diagnosed when the serum sodium level was ≤135 mmol/L, and, when present, its onset, frequency, and time course were monitored. Central DI includes both transient DI and permanent DI [8]; transient DI is diagnosed when hypotonic polyuria (40 mL/kg body weight daily) ensues soon after surgery and is usually self-remitting within a few days. Since permanent DI requires assessment of endocrine function several months after surgery, the incidence of DI in this study may only represent transient DI.

### 2.5. Follow-Up

All patients were followed up as outpatients for three months after surgery and then at annual or biannual intervals. Follow-up information for patients not attending our department was obtained by contacting the patients or their relatives by telephone or mail. Normalization was defined as a return of hormone levels to within the normal range without the requirement of any further treatment. Recurrence was diagnosed from the clinical features, hormonal function, or identification of residual tumor, based on the results of hormone and MRI examinations performed two years after surgery.

### 2.6. Statistical Analysis

Quantitative data are reported as the mean ± standard deviation (SD), and qualitative data are expressed as percentages. Quantitative and nonparametric data, such as mean age, time to diagnosis, and tumor size, were compared by Student's *t*-tests or Mann-Whitney *U* tests. Chi-square tests were used to compare qualitative data, including gender, invasiveness, the presence of pituitary apoplexy, tumor classification, remission rate, and recurrence rate. Statistical comparisons of endocrine outcomes at different follow-up times were adjusted for multiple testing using a Bonferroni correction. Differences between groups in endocrine outcome at the same follow-up time were assessed by Student's *t*-test. Correlations between variables were evaluated using Pearson or Spearman correlation coefficients. Analyses were performed using Statistical Package for Social Sciences Version 16.0 (Chicago, IL, USA). All statistical tests were two-sided, and *P* < 0.05 was considered statistically significant.

## 3. Results

### 3.1. Baseline Characteristics

A total of 84 patients were enrolled in the study, with a mean age of 33.60 ± 12.70 years (range: 17–75 years); more than half the patients were female (*n* = 59; 70.2%). The demographics and tumor characteristics of the two groups of patients are summarized in Tables 1 and 2.

Of the 84 patients enrolled, 48 (15 males and 33 females) were categorized into the CD group and 36 (10 males and 26 females) into the CD + PRL group. At the time of diagnosis, the patients in the CD + PRL group (mean age: 30.28 ± 14.23 years; range: 17–75 years) were significantly younger than those in the CD group (mean age: 36.08 ± 10.91 years; range: 23–65 years) (*P* = 0.037). Furthermore, maximal adenoma size was larger for patients in the CD + PRL group (mean diameter: 2.44 ± 1.32 cm; range: 0.6–6.0 cm) than for those in the CD group (mean diameter: 1.44 ± 1.05; range: 0.5–4.0 cm) (*P* < 0.001). The mean time to diagnosis did not differ significantly between the CD group (25.32 ± 37.98 months; range: 0.1–180 months) and the CD + PRL group (33.68 ± 34.96 months; range: 0.3–120 months), and there were also no significant differences in the incidences of invasiveness or pituitary apoplexy between the two groups. The patients were classified according to the degree of invasion of the cavernous sinus according to Knosp classification, and the two groups showed significant differences with the CD + PRL group showing a tendency for higher grades reflecting higher rates of invasion (*P* = 0.005).

### 3.2. Tumor Classification

In the CD group, 47.9% of the patients had microadenomas, and 52.1% had macroadenomas. The corresponding values for patients in the CD + PRL group were 5.6% and 94.4%, indicating that macroadenomas were more prevalent in the CD + PRL group than in the CD group (*P* < 0.001).

### 3.3. Preoperative Clinical Manifestations

The most commonly presenting clinical manifestations in the two groups were menstrual disorders, headaches, and dizziness (Table 3). Menstrual disorders and visual field defects were more common in patients in the CD + PRL group than in patients in the CD group (*P* = 0.027 and 0.021, resp.), whereas the incidences of progressive obesity and hypertension were much lower in patients in the CD + PRL group than in patients in the CD group (*P* = 0.009 and *P* < 0.001, resp.).

### 3.4. Endocrine Function before and after Surgery

Preoperative and postoperative serum cortisol and PRL levels were available for all patients (Table 4). Elevated preoperative levels of serum cortisol and PRL were detected in patients in the CD + PRL group, and these patients had lower cortisol levels and higher PRL levels than those in the CD group (*P* < 0.05 for both comparisons). The cortisol level of patients in the CD group decreased from 833.87 ± 235.75 nmol/L before surgery to 336.87 ± 267.56 nmol/L at 3 days, 320.64 ± 213.54 nmol/L at 3 months, and 332.01 ± 229.81 nmol/L at 12 months after surgery. The preoperative and postoperative PRL levels in the CD group were all within the normal range. In the CD + PRL group, the cortisol level decreased from 673.24 ± 65.53 nmol/L before surgery to 375.83 ± 187.51 nmol/L at 3 days, 385.24 ± 186.57 nmol/L at 3 months, and 368.33 ± 170.87 nmol/L at 12 months after surgery. Furthermore, the PRL levels of this group decreased from 233.63 ± 188.06 ng/mL before surgery to 72.63 ± 66.94 ng/mL at 3 days, 74.51 ± 62.58 ng/mL at 3 months, and 77.43 ± 70.38 ng/mL at 12 months after surgery. There were no significant differences in the postoperative cortisol levels between the CD and CD + PRL groups, but the postoperative PRL levels in the CD + PRL group remained markedly higher than those in the CD group.

**Figure 3:**
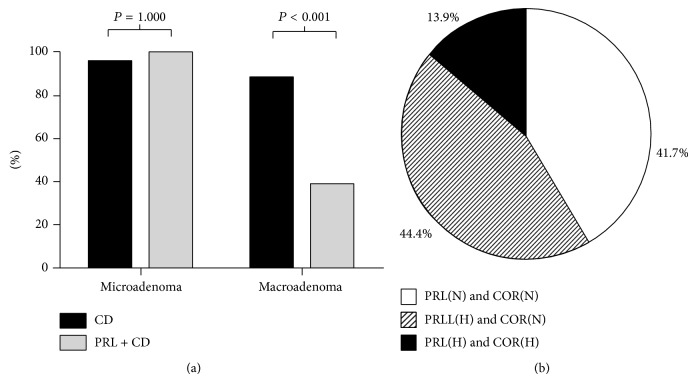
The rate of hormonal normalization in patients with Cushing's disease. (a) Comparisons of the normalization rates (%) after transsphenoidal surgery between microadenomas and macroadenomas, for patients in the CD and CD + PRL groups. (b) Distribution of the hormonal normalization rate in the CD + PRL group. “H” indicates hormone levels above normal after surgery; “N” indicates hormone levels in the normal range after surgery.

Among the patients with microadenomas, the normalization rates (based on the postoperative hormone levels at three months) in the CD and CD + PRL groups were 95.7% and 100%, respectively (Figure 3(a)). For patients with macroadenomas, the corresponding normalization rates were 88.0% and 38.2%, respectively. Overall, normalization of hormonal levels was achieved in 44 patients (91.7%) in the CD group, but only 15 patients (41.7%) in the CD + PRL group (*P* < 0.001). In the CD + PRL group, normalization of both cortisol and PRL levels occurred in 41.7% of the patients (Figure 3(b)).

### 3.5. Postoperative Complications

No deaths or major complications were observed, although some minor complications did occur, including transient DI and hyponatremia. Transient DI was observed in 26 of 48 patients (54.2%) in the CD group and in 18 of 36 patients (50.0%) in the CD + PRL group. Hyponatremia occurred in 50% of the patients in both groups, with nadir serum sodium levels in the CD group (113–134 mmol/L) similar to those in the CD + PRL group (109–130 mmol/L). There were no significant differences in the incidences of transient DI and hyponatremia between the two groups.

### 3.6. Follow-Up

The median postoperative duration of hospitalization was 5 days (range: 3–15 days). Overall, 90% of the patients were discharged by postoperative day 5. The median duration of follow-up for the entire group was 45 months (range: 13–121 months). Tumor recurrence was significantly less frequent in the CD group (4 patients, 8.3%) than in the CD + PRL group (13 patients; 36.1%) (*P* < 0.001). All recurrences arose within the first two years after surgery.

## 4. Discussion

Cushing's disease, caused by excessive ACTH secretion from tumorous pituitary corticotrophs, is a potentially life-threatening endocrine condition [9]. The first-line treatment for Cushing's disease is pituitary surgery to control excessive hormone production and improve pituitary function [10]. Cases of pituitary adenoma associated with increased production of both ACTH and PRL, causing apparent Cushing's disease and hyperprolactinemia, are extremely rare [5, 11]. The present study has evaluated the clinical features and endocrine and surgical outcomes, over a relatively long follow-up period, of a large series of patients who underwent transsphenoidal surgery for Cushing's disease with or without hyperprolactinemia.

The majority of our patients were diagnosed in their thirties and forties, with those in the CD + PRL group significantly younger at diagnosis than those in the CD group. In addition, our results revealed that the mean maximal diameter of the adenoma varied between the two groups, consistent with a previous report that patients with Cushing's disease alone usually present with small tumors [12]. The CD + PRL group also had a tendency for higher Knosp grades so the adenoma was more likely to have invaded the cavernous sinus space. Approximately half of the patients in the CD group were found to have microadenomas, and the other half were found to have macroadenomas, a finding similar to that of others [13]. Although recent advances in imaging technologies, including MRI, can detect relatively small lesions within the pituitary gland, these methods can localize microadenomas in only 60–80% of patients with Cushing's disease, since most of the lesions are very small [8, 14]. In our study, the average diameter of the adenoma was less than 5 to 6 mm, in agreement with previous investigations [8, 15]. We also found that nearly 95% of patients in the CD + PRL group had macroadenomas; this higher incidence may be related to the higher levels of PRL.

Menstrual disorders and visual field defects were more common in patients in the CD + PRL group (compared with the CD group), whereas the incidences of progressive obesity and hypertension were much lower. Hypersecretion of PRL has a variety of manifestations, including menstrual disorders in women [16], and our findings concurred with those of previous investigations [17]. Pituitary compression by a tumor may present with visual field defects; furthermore, patients in the CD + PRL group were much more likely to have macroadenomas, which are more commonly associated with the development of neurological deficits and visual disturbances. ACTH has antagonistic effects on protein and lipid metabolism that can lead to uncontrolled hypertension and other serious complications [18]. Overall, the clinical symptoms observed in our study agreed with those of previous reports in the literature [19–21]. Of the patients in the CD + PRL group who had hormonal symptoms, most were related to plurihormone production.

Elevated preoperative cortisol and PRL levels were detected in patients in the CD + PRL group, with PRL levels improving after surgery; this is consistent with the findings of Ratliff and Oldfield [22] and Wynne et al. [23]. We also found that patients in the CD + PRL group with high PRL levels often had a relatively low serum cortisol level. Yamaji and colleagues reported PRL elevation in 23% of patients with Cushing's disease [24], while Caufriez and coworkers found that 91% of patients with Cushing's disease had preoperative elevation of PRL levels that normalized after transsphenoidal surgery [25]. Although Wynne et al. reported that the predictive value of preoperative PRL levels in Cushing's disease was limited [23], we found that patients in the CD group were more likely to achieve normalization of endocrine function. It was notable that, in the CD + PRL group, the normalization rate for patients with macroprolactinomas was significantly lower than that for patients with microprolactinomas. Since complete resection of microadenomas was possible, it is perhaps not surprising that the normalization rates for patients with microadenomas exceeded 95% for both groups.

Our results, together with those of previous investigations, indicate that surgery is a highly effective treatment for Cushing's disease, and transsphenoidal surgery is considered the definitive treatment method [26]. Thus, the discovery of patients with a plurihormonal tumor depends on the hormonal levels as well as pathological identification [27, 28]. Plurihormonal adenomas are defined as those that secrete two or more hormones that differ in chemical composition, immunoreactivity, and biologic effects [29, 30]. In every patient in the CD + PRL group, a mixed tumor with ACTH- and PRL-positive cells was discovered by pathological investigations following surgery.

Transsphenoidal surgery represents a relatively safe procedure for resecting tumors underlying Cushing's disease. However, even in the most experienced hands, postoperative complications are unavoidable. The occurrences of postoperative transient DI and hyponatremia correlate with intraoperative manipulation of the pituitary stalk and posterior lobe, multiple incisions in the gland, and partial hypophysectomy [31]. Since pituitary exploration may damage the normal gland, gland manipulation is always kept to the minimum necessary to identify and remove the adenoma. The rate of postoperative transient DI reported in the literature varies widely [32, 33]. Although there was no significant difference in the incidence of DI between the two groups in our study, relatively higher rates of DI were observed in our patients overall, likely due to an increased awareness regarding this potential complication. There was also no significant difference in the incidence of hyponatremia between the two groups. Future prospective studies of the impact of transsphenoidal surgery on transient DI and electrolyte abnormalities are merited.

Wide variations in surgical outcomes and recurrence rates have been reported, depending on the tumor characteristics, the surgeon's experience, and the duration of the follow-up. The recurrence rate in the CD group was 8.3%, lower than that reported by others [34, 35], whereas it was 36.1% in the CD + PRL group. The lower recurrence rate in the CD group may reflect long-term remission. The elevated rate of recurrence in the CD + PRL group is an important finding, which suggests that careful long-term follow-up is needed in this subset of patients, even if surgery is considered successful.

Mixed ACTH and PRL adenomas often manifest themselves as coexisting Cushing's disease and prolactinoma [5]. Several hypotheses have been proposed to explain the coexistence of two or more hormones within an adenoma. First, neoplastic transformation caused by stimulation could result in different cell types [36]. Second, different hormones could be derived from distinct cell types within the borders of a single adenoma [4, 37]; for example, two separate tumors growing in a small space could become conjoined as a single mass.

Our study is not without its limitations. First, this was a retrospective cohort study in a single institution; prospective, multicenter studies are merited to extend our observations. Second, the follow-up period was relatively short for some patients, limiting the evaluation of the real recurrence rates. Hence, long-term postoperative follow-up of a similar cohort of patients is needed to better assess the effects of surgery and treatment outcomes.

## 5. Conclusion

In summary, the current study has compared characteristics between patients with both Cushing's disease and hyperprolactinemia and those with Cushing's disease alone. We found that patients in the CD + PRL group were characterized by younger age, larger tumor size, more clinical manifestations (including a higher incidence of menstrual disorders caused by PRL secretion), lower endocrine normalization rate, and more frequent recurrence rate, compared with patients with Cushing's disease alone. Our observations extend our knowledge of the characteristics of patients with Cushing's disease and hyperprolactinemia and may provide insights that will help to improve the diagnosis and treatment of the disease.

## Figures and Tables

**Table 1:** Preoperative demographic characteristics and tumor sizes of the patients in the two groups.

Variable	CD (*n* = 48)	PRL + CD (*n* = 36)	*P *
Age (years)	36.08 ± 10.91	30.28 ± 14.23	0.037
Time to diagnosis (months)	25.32 ± 37.98	33.68 ± 34.96	0.177
Tumor size (cm)	1.44 ± 1.05	2.44 ± 1.32	<0.001

Data are expressed as the mean ± SD. *P* values were determined using a Student's *t*-test or a Mann-Whitney *U* test, as appropriate. *P* < 0.05 was considered statistically significant.

**Table 2:** Gender, tumor invasiveness, and the presence of pituitary apoplexy in the two groups of patients.

Variable	CD (*n* = 48)	PRL + CD (*n* = 36)	*P *
Gender			
Male	15 (31.3%)	10 (27.8%)	0.461
Female	33 (68.8%)	26 (72.2%)	
Invasion			
+	11 (22.9%)	12 (33.3%)	0.289
−	37 (77.1%)	24 (66.7%)	
Apoplexy			
+	9 (18.8%)	6 (16.7%)	0.805
−	39 (81.3%)	30 (83.3%)	
Classification Knosp			
0	25 (52.1)	7 (19.4)	0.005
1	7 (14.6)	9 (25.0)	
2	6 (12.5)	7 (19.4)	
3	8 (16.7)	8 (22.2)	
4	2 (4.2)	5 (13.9)	

Data are expressed as *n* (%). “+” and “−” indicate the presence and absence, respectively, of the characteristic in the group. *P* values were determined using a chi-square test or a Mann-Whitney *U* tests. *P* < 0.05 was considered statistically significant.

**Table 3:** Preoperative clinical manifestations in the two groups of patients.

Symptom	CD (*n* = 48)	PRL + CD (*n* = 36)	*P *
Headache/dizziness	27 (56.3%)	24 (66.7%)	0.333
Progressive obesity	21 (43.8%)	6 (16.7%)	0.009
Vomiting	4 (8.3%)	4 (11.1%)	0.720
Visual impairment	19 (39.6%)	18 (50.0%)	0.341
Visual field defects	6 (12.5%)	12 (33.3%)	0.021
Menstrual disorders	16/28 (57.1%)	22/26 (84.6%)	0.027
Galactorrhea	10/28 (35.7%)	14/26 (53.8%)	0.180
Sexual dysfunction	4/10 (40.0%)	2/20 (10.0%)	0.141
Polyuria/polydipsia	12 (25.0%)	8 (22.2%)	0.767
Hypertension	40 (83.3%)	10 (27.8%)	<0.001
Diabetes mellitus	5 (10.4%)	6 (16.7%)	0.523

Data are expressed as *n* (%). *P* values were determined using a chi-square test. *P *< 0.05 was considered statistically significant.

**Table 4:** The levels of cortisol (nmol/L) and PRL (ng/mL) measured during follow-up of the patients in the two groups.

Group	Hormone	Before surgery	3 days after surgery	3 months after surgery	12 months after surgery	*F*	*P*
CD (*n* = 48)	Cortisol	833.87 ± 235.75^*^	336.87 ± 267.56	320.64 ± 213.54	332.01 ± 229.81	54.098	<0.001
	PRL	13.77 ± 6.87^△^	11.12 ± 6.71^△^	12.93 ± 6.57^△^	12.66 ± 6.20^△^	1.345	0.305

PRL + CD (*n* = 36)	Cortisol	673.24 ± 65.53	375.83 ± 187.51	385.24 ± 186.57	368.33 ± 170.87	30.713	<0.001
	PRL	233.63 ± 188.06	72.63 ± 66.94	74.51 ± 62.58	77.43 ± 70.38	18.64	<0.001

Data are expressed as the mean ± SD. ∗ indicates a significant difference in the cortisol level between the two groups at the same time point (*P* < 0.05). △ indicates a significant difference in the PRL level between the two groups at the same time point (*P* < 0.05). Each *P* value shown in the Table is for comparative analysis, within the CD or PRL + CD group, between values before surgery and those 3 days after surgery.

## References

[1] Egger J., Kapur T., Nimsky C., Kikinis R. (2012). Pituitary adenoma volumetry with 3D Slicer. *PLoS ONE*.

[2] Steffensen C., Bak A. M., Zøylner Rubeck K., Jørgensen J. O. L. (2010). Epidemiology of Cushing's syndrome. *Neuroendocrinology*.

[3] Lado-Abeal J., Rodriguez-Arnao J., Newell-Price J. D. C., Perry L. A., Grossman A. B., Besser G. M., Trainer P. J. (1998). Menstrual abnormalities in women with cushing's disease are correlated with hypercortisolemia rather than raised circulating androgen levels. *Journal of Clinical Endocrinology and Metabolism*.

[4] Mahler C., Verhelst J., Klaes R., Trouillas J. (1991). Cushing's disease and hyperprolactinemia due to a mixed ACTH- and prolactin-secreting pituitary macroadenoma. *Pathology: Research and Practice*.

[5] Sherry S. H., Guay A. T., Lee A. K., Whyte E. T. H., Federman M., Freidberg S. R., Woolf P. D. (1982). Concurrent production of adrenocorticotropin and prolactin from two distinct cell lines in a single pituitary adenoma: a detailed immunohistochemical analysis. *Journal of Clinical Endocrinology and Metabolism*.

[6] Lania A., Beck-Peccoz P. (2012). Pituitary incidentalomas. *Best Practice and Research: Clinical Endocrinology and Metabolism*.

[7] Knosp E., Kitz K., Steiner E., Matula C. (1991). Pituitary adenomas with parasellar invasion.. *Acta Neurochirurgica, Supplement*.

[8] Mampalam T. J., Tyrrell J. B., Wilson C. B. (1988). Transsphenoidal microsurgery for Cushing disease. A report of 216 cases. *Annals of Internal Medicine*.

[9] Barbot M., Albiger N., Koutroumpi S., Ceccato F., Frigo A. C., Manara R., Fassina A., Gardiman M. P., Scanarini M., Mantero F., Scaroni C. (2013). Predicting late recurrence in surgically treated patients with Cushing's disease. *Clinical Endocrinology*.

[10] Esposito V., Santoro A., Minniti G., Salvati M., Innocenzi G., Lanzetta G., Cantore G. (2004). Transsphenoidal adenomectomy for GH-, PRL- and ACTH-secreting pituitary tumours: outcome analysis in a series of 125 patients. *Neurological Sciences*.

[11] McNicol A. M. (1985). Current topics in neuropathology. Cushing's disease. *Neuropathology and Applied Neurobiology*.

[12] Tyrrell J. B., Wilson C. B. (1994). Cushing's disease. Therapy of pituitary adenomas. *Endocrinology and Metabolism Cinics of North America*.

[13] Kristof R. A., Schramm J., Redel L., Neuloh G., Wichers M., Klingmüller D. (2002). Endocrinological outcome following first time transsphenoidal surgery for GH-, ACTH-, and PRL-secreting pituitary adenomas. *Acta Neurochirurgica*.

[14] Doppman J. L., Frank J. A., Dwyer A. J., Oldfield E. H., Miller D. L., Nieman L. K., Chrousos G. P., Cutler G. B., Loriaux D. L. (1988). Gadolinium DTPA enhanced MR imaging of ACTH-secreting microadenomas of the pituitary gland. *Journal of Computer Assisted Tomography*.

[15] Atkinson A. B. (1991). The treatment of Cushing's syndrome. *Clinical Endocrinology*.

[16] Isik S., Berker D., Tutuncu Y. A., Ozuguz U., Gokay F., Erden G., Ozcan H. N., Kucukler F. K., Aydin Y., Guler S. (2012). Clinical and radiological findings in macroprolactinemia. *Endocrine*.

[17] Furuhata S., Kameya T., Otani M., Toya S. (1993). Prolactin presents in all pituitary tumors of acromegalic patients. *Human Pathology*.

[18] Hamilton D. K., Vance M. L., Boulos P. T., Laws E. R. (2005). Surgical outcomes in hyporesponsive prolactinomas: analysis of patients with resistance or intolerance to dopamine agonists. *Pituitary*.

[19] Fideleff H. L., Boquete H. R., Suárez M. G., Azaretzky M. (2009). Prolactinoma in children and adolescents. *Hormone Research*.

[20] Lania A. G., Ferrero S., Pivonello R., Mantovani G., Peverelli E., Di Sarno A., Beck-Peccoz P., Spada A., Colao A. (2010). Evolution of an aggressive prolactinoma into a growth hormone secreting pituitary tumor coincident with GNAS gene mutation. *Journal of Clinical Endocrinology and Metabolism*.

[21] Melmed S. (2008). Update in pituitary disease. *The Journal of Clinical Endocrinology & Metabolism*.

[22] Ratliff J. K., Oldfield E. H. (2000). Multiple pituitary adenomas in Cushing's disease. *Journal of Neurosurgery*.

[23] Wynne A. G., Scheithauer B. W., Young W. F., Kovacs K., Ebersold M. J., Horvath E., Parent A. D., Laws E. R. (1992). Coexisting corticotroph and lactotroph adenomas: case report with reference to the relationship of corticotropin and prolactin excess. *Neurosurgery*.

[24] Yamaji T., Ishibashi M., Teramoto A., Fukushima T. (1984). Hyperprolactinemia in Cushing's disease and Nelson's syndrome. *Journal of Clinical Endocrinology and Metabolism*.

[25] Caufriez A., Desir D., Szyper M., Robyn C., Copinschi G. (1981). Prolactin secretion in Cushing's disease. *The Journal of Clinical Endocrinology and Metabolism*.

[26] Biller B. M. K., Grossman A. B., Stewart P. M., Melmed S., Bertagna X., Bertherat J., Buchfelder M., Colao A., Hermus A. R., Hofland L. J., Klibanski A., Lacroix A., Lindsay J. R., Newell-Price J., Nieman L. K., Petersenn S., Sonino N., Stalla G. K., Swearingen B., Vance M. L., Wass J. A. H., Boscaro M. (2008). Treatment of adrenocorticotropin-dependent cushing's syndrome: a consensus statement. *The Journal of Clinical Endocrinology and Metabolism*.

[27] Kontogeorgos G., Kovacs K., Horvath E., Scheithauer B. W. (1991). Multiple adenomas of the human pituitary. A retrospective autopsy study with clinical implications. *Journal of Neurosurgery*.

[28] Parent A. D., Bebin J., Smith R. R. (1981). Incidental pituitary adenomas. *Journal of Neurosurgery*.

[29] Kovacs K., Horvath E., Asa S. L., Stefaneanu L., Sano T. (1989). Pituitary cells producing more than one hormone: human pituitary adenomas. *Trends in Endocrinology and Metabolism*.

[30] Ho D. M., Hsu C. Y., Ting L. T., Chiang H. (2001). Plurihormonal pituitary adenomas: immunostaining of all pituitary hormones is mandatory for correct classification. *Histopathology*.

[31] Olson B. R., Gumowski J., Rubino D., Oldfield E. H. (1997). Pathophysiology of hyponatremia after transsphenoidal pituitary surgery. *Journal of Neurosurgery*.

[32] Jho H.-D. (2001). Endoscopic transsphenoidal surgery. *Journal of Neuro-Oncology*.

[33] Cappabianca P., Cavallo L. M., Colao A., de Divitiis E. (2002). Surgical complications associated with the endoscopic endonasal transsphenoidal approach for pituitary adenomas. *Journal of Neurosurgery*.

[34] Kim J. H., Shin C. S., Paek S. H., Jung H. W., Kim S. W., Kim S. Y. (2012). Recurrence of Cushing's disease after primary transsphenoidal surgery in a university hospital in Korea. *Endocrine Journal*.

[35] Alexandraki K. I., Kaltsas G. A., Isidori A. M. (2013). Long-term remission and recurrence rates in Cushing's disease: predictive factors in a single-centre study. *European Journal of Endocrinology / European Federation of Endocrine Societies*.

[36] Kovacs K., Horvath E., Stefaneanu L., Bilbao J., Singer W., Muller P. J., Thapar K., Stone E. (1998). Pituitary adenoma producing growth hormone and adrenocorticotropin: a histological, immunocytochemical, electron microscopic, and in situ hybridization study. *Journal of Neurosurgery*.

[37] Scheithauer B. W., Horvath E., Kovacs K., Laws E. R., Randall R. V., Ryan N. (1986). Plurihormonal pituitary adenomas. *Seminars in Diagnostic Pathology*.

